# Camel viral diseases: Current diagnostic, therapeutic, and preventive strategies

**DOI:** 10.3389/fvets.2022.915475

**Published:** 2022-08-11

**Authors:** Mahmoud Kandeel, Abdullah I. A. Al-Mubarak

**Affiliations:** ^1^Department of Biomedical Sciences, College of Veterinary Medicine, King Faisal University, Al-Hofuf, Saudi Arabia; ^2^Department of Pharmacology, Faculty of Veterinary Medicine, Kafrelsheikh University, Kafrelsheikh, Egypt; ^3^Department of Microbiology, College of Veterinary Medicine, King Faisal University, Al-Hofuf, Saudi Arabia

**Keywords:** viral diseases, camel, vaccine, drug, control

## Abstract

Many pathogenic viruses infect camels, generally regarded as especially hardy livestock because of their ability to thrive in harsh and arid conditions. Transmission of these viruses has been facilitated by the commercialization of camel milk and meat and their byproducts, and vaccines are needed to prevent viruses from spreading. There is a paucity of information on the effectiveness of viral immunizations in camels, even though numerous studies have looked into the topic. More research is needed to create effective vaccines and treatments for camels. Because Camels are carriers of coronavirus, capable of producing a powerful immune response to recurrent coronavirus infections. As a result, camels may be a suitable model for viral vaccine trials since vaccines are simple to create and can prevent viral infection transfer from animals to humans. In this review, we present available data on the diagnostic, therapeutic, and preventative strategies for the following viral diseases in camels, most of which result in significant economic loss: camelpox, Rift Valley fever, peste des petits ruminants, bovine viral diarrhea, bluetongue, rotavirus, Middle East respiratory syndrome, and COVID-19. Although suitable vaccines have been developed for controlling viral infections and perhaps interrupting the transmission of the virus from the affected animals to blood-feeding vectors, there is a paucity of information on the effectiveness of viral immunizations in camels and more research is needed. Recent therapeutic trials that include specific antivirals or supportive care have helped manage viral infections.

## Introduction

Camels are known for their ability to survive and cope in the harshest environments (1), and millions of people living in the pastoral regions of central Asia, Africa, and several Western areas require camels to support their income and daily life (2–4). Because of the camel's importance, it is crucial to investigate the occurrence of viral infections in camel species, which constitute a major health and zoonotic hazard.

Historically, dromedary camels were thought to be especially resistant to infectious diseases to which most domestic animals were susceptible. This assumption has since been disproven, as camels have demonstrated susceptibility to many viral infections and can act in their transmission (5). Initially, camels were known to be the primary species to contract camelpox, which was host-specific and not zoonotic. The zoonotic potential of camel infections was first observed in 2011 in India when three people were diagnosed with disease transferred from camels (6). Today, camels are known to be carriers of several infectious diseases, including peste des petits ruminants (PPR), African horse sickness, Rift Valley fever (RVF), bluetongue (BT), and West Nile disease. In 2017, camels in Kenya exhibited the highest seroprevalence (99% after seroprevalence) of the influenza D virus (IDV), which causes respiratory infections primarily in cattle, sheep, goats, and other livestock species (7). Influenza C virus (ICV) has also been detected in dromedary camels in Kenya (8). Another cattle-specific viral infection is bovine viral diarrhea (BVD), caused by bovine viral diarrhea virus (BVDV), of which there are two primary variants, BVDV1 and BVDV2 (9). BVDV is known to infect a wide range of wild and domestic animals, including sheep, deer, swine, and camels. The susceptibility of old-world (dromedary and Bactrian) and New World camelids (llama, alpaca, guanacos, and vicunas) to BVDV varies, and further investigation is needed (10). Furthermore, the susceptibility of camels to epizootic hemorrhagic disease (EHD) requires further exploration.

**Figure 1:**
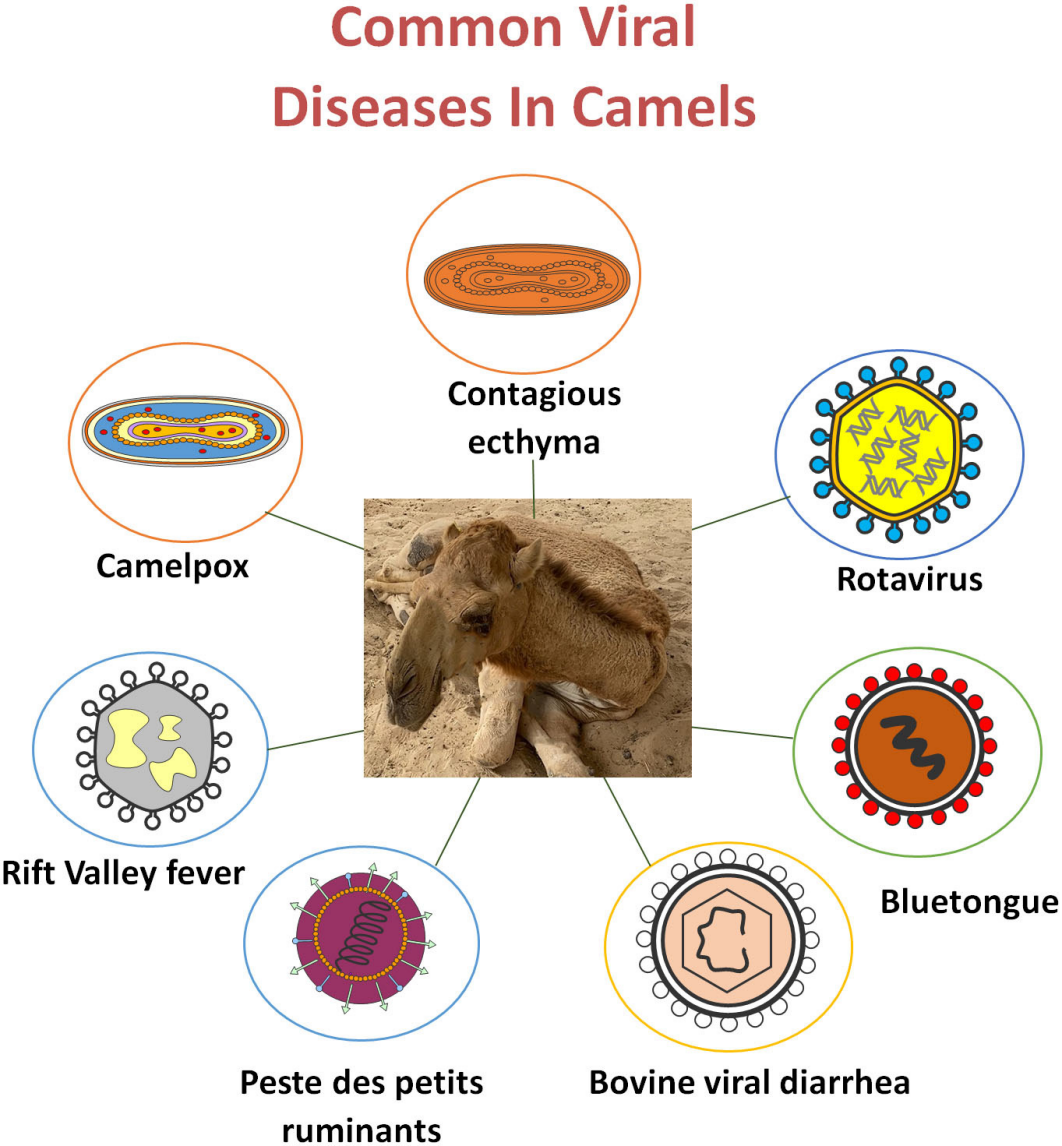
Common viral diseases in camels.

The current review describes the importance of viral infectious diseases in camels. We provide available data on the treatment and prevention approaches for camel viral diseases, including camelpox, RVF, BVD, PPR, BT, Middle East respiratory syndrome (MERS) coronavirus infection, and severe acute respiratory syndrome (SARS) coronavirus (Figure 1).

## Infection with poxviruses in camels

### Camelpox

#### Epidemiology and diagnostics

Camelpox is an extremely infectious skin disorder and the most common infectious viral illness of camels, occurring in most regions where camel farming is common. The camelpox virus (CMLV; genus *Orthopoxvirus*, family *Poxviridae*), the cause of this infectious disease, is closely related to the variola virus. The genes associated with viral replication activities and most of those important in additional host-related processes are identical in the two viruses (11).

Symptoms of camelpox are fever, skin lesions, and lymph node inflammation. Pox lesions of different stages may form, most notably on the face, throat, and near the tail. The disease may be diagnosed based on clinical symptoms, although camel contagious ecthyma and camel papillomatosis induce similar symptoms (12). Multiple diagnostic methods are available, including transmission electron microscopy (TEM), the most rapid diagnostic tool for detecting the characteristic, brick-shaped orthopoxvirus in tissue samples or skin lesions (13). Immunohistochemistry can also be informative. PCR may be used to confirm the presence of viral nucleic acid, and DNA restriction enzyme testing can be used to identify specific strains of the CMLV (13, 14).

#### Prevention and control

The infection, like smallpox in humans, may be controlled by separating diseased camels and injecting the remainder with the standard vaccinia virus vaccine or the recently developed CMLV vaccine, available live attenuated and inactivated (15). A booster vaccine dose is recommended for young camelids inoculated before 6–9 months. The inactivated vaccine can be injected yearly (16), and the live attenuated vaccine provides long-term protection.

A live attenuated candidate vaccine was produced in Sudan using a local strain of CMLV and assessed in a small-scale field study for safety and efficacy in experimental camels (17). Most tests revealed that the proposed vaccine is effective, safe, and can control the infection. Most vaccines are produced from the CMLV strains Ducapox 298/89, Jouf-78, VD47/25, and CMLV-T8. The attenuated Jouf-78 strain has been found to provide complete protection against CMLV (17). A new vaccine developed from serial multiplication of the KM-40 virus strain on the chorioallantoic membranes of 11-day-old embryonated chicken eggs has the potential to protect against camelpox in Old World camelids (*Camelus dromedaries* and *Camelus bactrianus*) (15).

#### Treatment

General non-specific treatment for infected camels is the administration of 10 mg/kg oxytetracycline and 0.2 mg/kg meloxicam for 5 days (18). A spray containing gamma benzene hexachloride, proflavine hemisulphate, cetrimide, eucalyptus oil, turpentine oil, and neem oil can also be used for wound therapy and fly control (18). Other ethnopharmacological applications are also widely used to treat camelpox (19). In humans, cidofovir would likely be beneficial in the treatment and short-term prevention of smallpox and kindred poxvirus infections, as well as the treatment of vaccinia sequelae in immunocompromised individuals (20). Cidovir and its acyclic nucleoside phosphonate derivatives have shown promising therapeutic potency against camelpox (21).

### Camel contagious ecthyma

#### Epidemiology and diagnostics

Camel contagious ecthyma (CCE) is a highly contagious viral disease with a 38% fatality rate that mostly affects young camels, causing calf debility. Caused by a poxvirus (genus *Parapoxvirus*, family *Poxviridae*), CCE is distinguished by pustular lesions around the mouth, lips, and buccal cavity, and head swelling (22). The disease is distinguished by a rapid onset and lesion development. PCR targeted to the RPO30 gene is the most often used assay for samples from skin scrapings or pustules (23). Negative contrast electron microscopy, immunofluorescence, and immunoperoxidase assays are also employed.

#### Prevention and control

Vaccination using CCE virus-containing material appears promising. However, immunization with vaccinia virus and a vaccine against infectious ecthyma in sheep and goats did not protect camels from infection (24).

#### Treatment

Traditional therapies include cauterization of regional lymph nodes, the application of sesame oil and heated milk, and plant tar. Topical or systemic broad-spectrum antibiotics for 3–5 days prevent subsequent bacterial infection. Antipyretics, antihistamines, and multivitamins are also helpful in minimizing the consequences of the infection (22). Administering the preferred NSAID flunixin (1 mg/kg) intramuscularly once daily may be recommended (25).

## Rift valley fever

### Epidemiology and diagnostics

The RVF is a severe mosquito-borne viral infection [genus *Phlebovirus*, family *Bunyaviridae* [26]] that affects animals (lambs, camels, and cattle) as well as humans in the Arabian Peninsula and Sub-Saharan Africa. Camelids have frequently been linked with RVF epidemics in East Africa and Egypt. RVF produces severe sickness in camels, with fever, weakness, abnormalities, infertility, and an increased death rate, especially among younger camels. Signs and symptoms of RVF are ocular discharge, tongue hemorrhages, and trough edema (26).

Reverse transcription-PCR (RT-PCR) can identify the virus in the blood (during infection) and tissues. The presence of RVFV is also confirmed by an enzyme-linked immunoassay (ELISA), which shows the presence of IgM autoantibodies that appear rapidly as an effective response to an acute infection and IgG antibodies which last for many years (27).

### Prevention and control

Many types of vaccines are available. Because of the necessity for several doses, inactivated or killed vaccines are unsustainable for regular field immunization. One of the oldest and most commonly used vaccines for managing RVF in Africa is the modified live Smithburn vaccine, which requires only a single dosage; however, it has been linked to birth abnormalities and miscarriages in pregnant calves, and it may only provide camels modest protection against RVF infection (28).

MP-12, a live-attenuated vaccine, has shown encouraging results in laboratory testing (29). Moreover, the live-attenuated Clone 13 vaccine has been approved for use in South Africa. Alternative immunizations based on recombinant molecular structures are now being investigated, with promising results. Camels showed a robust and long-lasting neutralizing immune response after receiving a single dose of the live CL13T RVF vaccine, demonstrating that the product is safe to use with no notable negative effects in the vaccinated camels (30).

### Treatment

Tilorone *in vitro* inhibited both the vaccine (MP-12) and the pathogenic (ZH501) strains of RVFV at low micromolar concentrations. In a mouse model, tilorone treatment significantly improved the survival outcomes of BALB/c mice exposed to RVFV ZH501. An 80% survival rate was achieved when 30 mg/kg/day was administered immediately after infection. One day after infection, 30% of animals administered tilorone at 45 mg/kg/day survived (31). Oral favipiravir (200 mg/kg/day) prevented death in ≥60% of hamsters challenged with RVFV when administered within 1 or 6 h of exposure and decreased initial RVFV titers in serum and tissues. Ribavirin (75 mg/kg/day) relieved some symptoms of peracute RVFV illness (32).

While pre-treatment with glucan was useful in identifying antiviral medicines to prevent RVFV, the most promising results were observed for the interferon inducer polyriboinosinic–polyribocytidylic acid complex with poly-l-lysine and carboxymethylcellulose [poly(ICLC)]. Ribavirin and poly(ICLC) have demonstrated the ability to prevent sickness in hamsters. Rhesus monkeys infected with RVFV had lower viremia after receiving ribavirin (50 mg/kg loading dose, then 10 mg/kg every 8 h for 9 days). Ribavirin also reduced viral production in infected cell cultures (33). Intramuscular administration of recombinant leukocyte A interferon and Sendai virus-induced human leukocyte interferon to RVFV-infected rhesus monkeys prevented viremia and hepatocellular damage (34). Curcumin was shown to be effective against highly virulent ZH501 and inhibited viral multiplication in infected animals' livers (35). Several other effective drugs against RVFV include suramin, sorafenib, sorafenib, rapamycin and 5,6-dimethoxyindan-1-one (36).

## Peste des petits ruminant

### Epidemiology and diagnostics

The PPR is an infectious disorder of sheep and goats that has unexpectedly reappeared and is now extensively dispersed throughout most of the Middle East, Africa, and Asia. Anorexia, diarrhea, oculonasal secretion, severe pyrexia, necrotic stomatitis, ulcerative, and respiratory failure are common symptoms of this infection. The virus that causes the infection is the peste des petits ruminants virus [PPRV; genus *Morbillivirus*, family *Paramyxoviridae* (37)]. Camels were not previously recognized as potential PPR hosts until a study in Egypt demonstrated positive serum reactivity in Sudanese camels (38). The first PPR epidemic in camelids was described in 1996 as a highly infectious respiratory illness with significant morbidity and low mortality rates. In 2004, an epidemic of a similar illness occurred in eastern Sudan, followed by outbreaks in Somalia and Kenya. Although the reports from Kenya and Somalia did not identify the causative agent, researchers in Sudan identified it as a PPR virus (39).

Pathological lesions and specific identification of viral antibodies or antigens in medical samples by different serologic tests and molecular assays may be used to diagnose the condition. Immunocapture ELISA and RT-PCR are common diagnostic methods.

### Prevention and control

Live-attenuated vaccines with long-lasting protection against PPRV are currently available. The first homologous PPR vaccine was developed using a live attenuated Nigerian strain (Nig 75/1) that exhibited solid protection for 3 years after 63 passes in Vero cells (40). Several immunization studies performed between 1989 and 1996 demonstrated the effectiveness of this vaccine. Under field conditions, the vaccine proved safe and induced protection in 98% of vaccinated camels. Following outbreaks with virulent PPRV strains, the vaccinated camelids did not develop an infection; therefore, this vaccine is currently used globally (Middle East, Africa, and southern Asia) for efficient PPR control. Sungri/96, PPRV Arasur/87, and CBE/97 stains are available as live attenuated PPR vaccines (41).

### Treatment

Out of 42 goats, 38 (93.23%) recovered after receiving an antibiotic and anti-inflammatory protocol of ampicillin trihydrate, colistin sulfate, and dexamethasone acetate IM once daily for 3 days, and metronidazole 500 mg/35 kg body weight orally three times daily for 3 days (42). Long-acting tetracyclines and topical gentian violet helped resolve the PPR lesions within 3 weeks (43). The antiparasitic endectocide ivermectin inhibited PPRV replication *in vitro*, suggesting that it might be used as a repurposed antiviral drug against PPRV (44).

## Bovine viral diarrhea

### Epidemiology and diagnostics

The BVDV is one of the numerous pestiviruses that infect domesticated and wild ruminants, camelids, and pigs worldwide. For cattle farmers, infection results in commercial losses due to lower growth weight, reduced milk supply, reproductive losses, and mortality. Aerosol transmission is considered the most effective mode of BVDV transmission in camelids. The virus is primarily transmitted by inhalation or ingestion (45), is present in all bodily fluids and excretions, and can transfer from mother to fetus. Fever, mouth ulcers, anorexia, diarrhea, abortion, general poor health, and birth abnormalities are some possible symptoms (46).

One-time testing cannot provide a definitive diagnosis of chronic BVDV infection in camelids. Although the BVDV antigen ELISA test is used to diagnose chronic BVDV infections in bovines, it is unknown whether a comparable interpretation of the results in camelids would be valid. As a result, chronic infections in camelids should be determined by virus identification *via* PCR or viral isolation in samples collected over 3 to 4 consecutive weeks (47).

### Prevention and control

No BVDV vaccine is currently approved for use in camelids, although several vaccines are available for use in cattle. Vaccines cannot prevent infection, but they do lessen the clinical symptoms of illness (48). Vaccinating camelids is not currently recommended, pending further studies. Inappropriate vaccination may prevent accurate diagnostic testing and the capacity to identify infected camelids.

Maintaining a sealed herd, establishing strong biosecurity standards for all entering animals (recommended for many viral infections, such as coronavirus), and inspecting open herds regularly may all help to limit the infection frequency (49).

Non-pregnant female alpacas were challenged *via* nasal and ocular injection of BVDV type 1b strain 25 days after immunization with a modified BVDV vaccine (50). The animals experienced no adverse effects. The modified-live BVDV Singer strain type 1 and BVDV 125c type 2 vaccines (Vista 3SC) were administered intravenously at a dosage of 2 ml, as directed for calves. The type 1b BVDV test strain, isolated from a chronically infected alpaca, was not detected in the immunized alpacas but was identified in the two unvaccinated controls (51).

Inactivated vaccines, by contrast, are safer and may provide adequate protection. Vaccines based on ncp BVDV strains have been developed. Authors of a recent study examining new world camels detected seroconversion following three injections with an inactivated virus preparation (52).

### Treatment

In cell culture, IFN-α substantially inhibited viral development. In the presence of more than 103 units/ml of IFN-α, the replication of two cytopathogenic and two non-cytopathogenic strains of BVD was inhibited. The addition of TNF-α or TNF-β to IFN-α did not influence the suppressive effect (53). Huang qin Zhizi, a Chinese herbal medication, proved beneficial in treating BVD. According to network and pathway analysis, the medication suppressed inflammation and increased host immune responses to BVD infection (54).

## Bluetongue disease

### Epidemiology and diagnostics

BT is an infectious disease caused by the bluetongue virus (BTV; genus *Orbivirus*, family *Reoviridae*) transmitted by biting midges of *Culicoides* spp. It primarily affects ruminant and camelid animals. Symptoms of this infection are more common in sheep, whereas asymptomatic BT infections are more prevalent in cows, goats, and camelids (55). Twenty-seven variants of BTV have thus far been identified. BTV 1–24 strains are recognized as classical, while BTV 25–27 are atypical. BT is spread worldwide, including the Americas, Africa, southern Asia, and northern Australia (56). As BT outbreaks profoundly affect the international trade of animals and animal products, the World Organization for Animal Health (OIE) listed BT as a communicable disease. The animal death rate, illness, infertility, and decreased growth rate result in huge direct economic losses, while trade reduction and medical costs cause indirect losses (57).

Although alpacas and llamas are prone to BTV outbreaks, they are asymptomatic. However, they act as carriers because BTV infection lasts for several days in those species. Camels are considered low-risk animals for BTV; however, some studies have reported BTV infection in camels, such as BTV infection in an alpaca (*Vicugna pacos*), llamas, and BT epidemic in Europe (58–60). Competitive ELISA, real-time PCR, and agar gel immunodiffusion tests are reportedly reliable and efficient BTV diagnostic tests recommended by OIE for international trade purposes (61).

### Prevention and control

Subunit and viral vector-based vaccines have been developed for BTV infection and are used in ruminants (62–64). These vaccines are not approved for South American camelids, although many experts advocate their use (65).

### Treatment

BTV is resistant to ether, chloroform, and sodium deoxycholate but sensitive to trypsin treatment. The virus is stable within a pH range of 6–8. Below a pH of 6, BTV was rendered inactive at 37°C (66). Levamisole, an anthelmintic drug, stimulated a significant immune response to the BT vaccine when administered in repeated doses of 2.5 mg/kg before immunization (67). *Tarantula cubensis* extract (Theranekron) showed promising efficacy in treating oral BT lesions in cattle when combined with tetracycline and flunixin meglumine (68). Re-epithelization and recovery to normal body temperature occurred 24 h after Theranekron administration, with the treatment group recovering faster than the control group.

## Middle East respiratory syndrome

### Epidemiology and diagnostics

In 2013, MERS was first identified as a human disease caused by an emerging coronavirus (MERS-CoV) mainly found in the Middle East (69–71). Multiple subsequent studies suggested that severe MERS can also be transmitted zoonotically from various animals to humans.

In 2013, researchers conducted a case study of a 43-year-old retired military veteran experiencing initial symptoms of shortness of breath, fever, rhinorrhea, and malaise, which worsened over time. The patient's personal history indicated that he owned a herd of nine camels and had been visiting the camels in their barn every day for 3 days before the onset of symptoms. One of the patient's acquaintances, who also visited the camels with the patient, reported to the doctors that four of the eight camels were suffering from a serious illness with evident nasal discharge. After admission to the hospital, the patient's condition deteriorated, and he eventually died. MERS-CoV was detected in nasal swab samples from both the camel and the patients. Subsequent genetic sequencing revealed similar sequences from the camel and the patient, and serological testing revealed that the camel had been exposed to MERS-CoV prior to the patient's illness, implying that the infection was passed from the sick camel to the person (72).

### Prevention, control, and treatment

Small chemical molecules, previously developed antivirals, and antiviral peptides have been used to treat MERS-CoV infection (73–79). Despite several human studies, no consensus has been reached on the best treatment for MERS-CoV (80). Several clinical trials and *in vitro* and *in vivo* studies have been attempted to identify an effective medication. The regimens investigated have included ribavirin for 5 days plus INF α-2b (81), lopinavir/ritonavir PO + ribavirin PO + PEG-INF (82), mycophenolate mofetil (83), small molecules, and fusion peptides (76, 78).

## COVID-19

Coronavirus disease 2019, COVID-19, was declared a global pandemic by the World Health Organization in 2020. COVID-19 is caused by severe acute respiratory syndrome coronavirus 2 (SARS-CoV-2; genus *Deltacoronavirus*, family *Coroniviridae*), a single-stranded RNA virus. Over six million people worldwide have died from the disease (84). Camels are known to be reservoirs and carriers of various coronavirus strains, and as a result, they can produce a strong immune response against recurrent coronavirus infections. Given that they are the known carriers of MERS-CoV, their immunity can be used to develop immunogenic responses against recurrent coronavirus strains (85). Lactating female camel carriers of the dormant form of MERS-CoV can produce a strong immune response against the stated coronavirus strain by producing camel-specific IgG antibodies. These antibodies can be found in the serum and milk of lactating female camels (86). The preparations of these camel antibodies can act as a source for camel vaccines to prevent SARS-CoV-2 infections. In a recent study, camel serum demonstrated cross-neutralizing activity against SARS-CoV-2. These camels were seropositive for MERS-CoV, but antigens were not detected. This observation implies that camels could be a source of specific antibodies for controlling COVID-19 (87).

The camel antibodies and nanoantibodies have been shown to elicit a strong immunogenic response and efficient neutralizing of viral antigens. Even at extreme temperatures (80°C), they can neutralize the infectious antigen by presenting on the surface and within the infected cell (88–90). Before directly developing new human vaccines, it is essential to determine the intermediate hosts responsible for MERS-CoV infection in humans. SARS-CoV-2 has been isolated from various animals, including cats, raccoons, rats, and civets, indicating that all of these animals can act as intermediate hosts. Bats were originally also thought to be important intermediate hosts for various coronaviruses. Animal vaccines are easier to design and validate than human vaccines because fewer validating steps and trials are required. The primary reason for vaccinating intermediate hosts is that their vaccine is simple to develop and can prevent viral infection from spreading from animals to humans.

The relationship between camels and SARS-CoV-2 extends beyond the infection of camels to an era of including camels in COVID-19 control. A library of nanobodies (Nbs) produced from immunized camels has been created. It was discovered that seven Nbs had a high affinity for at least eight SARS-CoV-2 RBD mutants. Among these candidates, Nb11-59 demonstrated the most neutralizing activity while maintaining a high level of stability, indicating that Nb11-59 may be a potential therapeutic molecule for an inhalation COVID-19 therapy (91). Several Nbs that bind to the SARS-CoV-2 RBD has been identified from vaccinated camelids. NIH-CoVnb-112, the principal treatment candidate, demonstrated strong affinity in monomeric form and inhibited the interaction between SARS-CoV-2 and ACE2 (92).

## Camel rotavirus

### Epidemiology and diagnostics

Rotavirus (RV) has been found in New World and Old World camels (93). The first African camel rotavirus strain was reported in 2014 (94). Some portions of the strain were related to human–animal reassortant rotaviruses. Multiple reassortment events between mammalian strains resulted in strain MRC-DPRU447. In a camel calf diarrhea survey in Sudan using group A rotavirus antibody, 53% showed high antibody titers (95). ELISA and the observed characteristic wheel-like morphology of rotavirus particles by electron microscopy were among the diagnostic procedures (96, 97). When delivered orally to calves and pigs in the field, live attenuated rotavirus vaccines were ineffective due to colostral antibodies that hindered vaccine virus multiplication. The presence of rotavirus antibodies in colostrum prompted the development of maternal rotavirus vaccination techniques to improve lactogenic immunity and transfer passive antibodies to the neonate *via* colostrum and milk (98).

### Prevention, control, and treatment

Rotavirus is the leading cause of death in children under the age of five. Camel milk is indicated in this case because it contains anti-rotavirus antibodies (99). The RV treatment strategy in humans depends mainly on rehydration, adjunctive therapy and probiotics (100). There is mounting evidence that certain probiotics can be used as an adjuvant to rehydration therapy. *Lactobacillus rhamnosus* GG (LGG), given at a daily dose of 10 billion colony-forming units per day, has been shown to lessen the length of diarrhea in individuals with rotavirus gastroenteritis. In treating rotavirus gastroenteritis in young children, a nitazoxanide antibiotic demonstrating activity against anaerobic bacteria, protozoa, and viruses enabled considerable decreases in time to symptom resolution (100). Racecadotril has been used as an antidiarrheal drug, decreasing the gastric transit time of RV.

## Other viruses in camels

Other viruses, such as rabies, have been detected in camels (101). Antibodies against several other viruses were detected in the serum of camels without specific fatalities, such as those causing African horse sickness, parainfluenza, akabane, foot and mouth disease (type O), and rinderpest (101).

## Surveys and vaccine trials against virus diseases in camels

Camels have been subjects of special interest in multiple seroprevalence surveys to detect viral diseases (Table 1). Antibodies to RVFV and CMLV have been detected in camels in various African and Asian nations, highlighting the potential influence of the zoonotic role of animals in disease transmission. In 2021, BVDV and PPRV were recorded for the first time in alpacas in northern China (113). In general, camels have been found to have a lower incidence of BDVV than other species. Notably, the BVD virus was found to be more prevalent in camels bred in the presence of other animals than in camels raised alone in camel barns (116). This observation implies that camels have a decreased overall susceptibility or self-limited BVDV infection. BT has been a less common viral disease among camels, although it is widespread in other species in the survey areas in the survey areas (119). The unexpectedly high frequency of MERS-CoV infection in camels is not linked with obvious clinical symptoms with the presence of high antibody titers. Camels may, as a result, defend themselves from the closely similar SARS-CoV-2. There has been no evidence of SARS-CoV-2 infection in camels. Vaccine trials against MERS-CoV based on DNA (122) or poxvirus vector vaccine (123) have shown promising results in camels. The recently developed antiviral vaccines and their efficacy are briefly summarized in Table 2.

**Table 1:** Summary of screening surveys conducted to evaluate the seroprevalence of various viruses in camels.

**Viral infection**	**Survey purpose**	**Targeted animal**	**Study finding**	**Region**	**Year**	**References**
Rift Valley fever virus (RVFV) infection	To evaluate the presence of RVFV antibodies among camels in Nigeria and the associated risk factors.	Dromedary Camel	Further investigation to unravel the zoonotic transmission potential to pastoralists and other animal species is pertinent.	Northern Nigeria	2021	(102)
	Analysis of 120 camel serum samples from northern Kenya to establish seropositivity rates of the Rift Valley fever (RVF), brucellosis and Q fever.	Dromedary camels	High seropositivity rates which indicate the endemicity of these pathogens among camel populations.	Kenya	2021	(103)
	Evaluation of seroprevalence of RVF through ELISA test	Camels + cattle	High serological prevalence of RVF in camels and cattle.	Southern Mauritania	2013	(104)
	A risk-based serological survey was performed to assess the prevalence of RVF through multispecies ELISA test.	Camels	As no RVF outbreaks have been reported in Tunisia and this survey study verified the absence of RVF in farm animals till January 2018.	Tunisia	2018	(105)
	A cross-sectional based was designed to find seroprevalence and analyze RVF-associated risk factors in camels slaughtered in Nigeria, through ELISA test.	*Camelus dromedarius*	Camels presented for slaughter at the Maiduguri abattoir, Nigeria have evidence of exposure to the RVF virus and thus can act as a source of RVF transmission.	Nigeria	2021	(106)
Camelpox	The isolation and molecular identification of live Camelpox virus from skin 12 months after the beginning of clinical indications.	Dromedary camels	There is a possibility of reinfection of some recovered camels is a method by which CMLV might ensure its survival in previously infected/vaccinated groups.	Saudi Arabia	2012	(107)
	Confirmation of spread of Camelpox Virus from Dromedary Camel to Human through genomic sequencing	Dromedary camels	Epidemiological data and the genomic sequences of CMLV from infected camels and humans suggested the zoonotic transmission of CMLV from camels to humans.	Sudan	2014	(108)
	Investigation of the clinicopathological changes related to camelpox outbreak in a dromedary camel herd *via* molecular characterization.	Camels	This disease mostly affects younger animals.	India	2017	(109)
	Evaluation of outbreak of a Systemic Form of Camelpox in the United Arab Emirates	Dromedary camels	The virulence of the virus is dependent on risk factors such as age, overall fitness, management, and environment.	United Arab Emirates	2021	(110)
Peste Des Petits Ruminants (PPR) Virus Infection	Investigation of PPRV infection prevalence in Egypt	Small Ruminants and Camels	PPRV is prevalent in Egypt, causing epidemics in its main host including small ruminants but no spread to camels is observed.	Egypt	2018	(111)
	Experimental inoculation with a pathogenic PPRV strain from lineage IV was used to test camel susceptibility for PPRV.	Young dromedary camels	Dromedary camels are not sensitive to PPRV after infection by an extremely pathogenic strain.	Morocco	2015	(112)
	Assessment of the seroprevalence of BVDV and PPRV antibodies in alpacas.	Alpacas	This was the 1st study that reported the BVDV and PPRV seroprevalence in alpacas in China.	Northern China	2021	(113)
	Evaluation of seroprevalence of PPRV through ELISA and virus neutralization test.	Camels and Cattle	No prevalence of PPRV in camels and while PPRV had 12% prevalence in cattle	Mauritania	2013	(104)
	Using the haemagglutination (HA) test, evaluation of the prevalence and serological proportion of PPRV in camels at a slaughterhouse	Young camels	PPRV could be found in camels' pneumonic lungs, suggesting its role in camel pneumonia.	Sudan	2021	(114)
Bovine Viral Diarrhea (BVD) infection	Antibody competitive ELISA and antigen detecting ELISA tests were used to determine the prevalence of chronic BVDV infection.	Camels + Cattle	Both antigen and antibody testing revealed a high prevalence of BVD virus in cattle as compared to camels.	Ethiopia	2021	(115)
	Assessment of the seroprevalence of BVDV and PPRV antibodies in alpacas	Alpacas	This was the 1st study that reported the BVDV and PPRV seroprevalence in alpacas in China	Northern China	2021	(113)
	Antigen and antibody ELISA tests were used to detect bovine viral diarrhea virus (BVDV), bovine in four distinct geographical locations of Turkey.	Dromedary camels	BVD virus infection was higher in camels from the herd raised with other ruminants as compared to those camels raised alone.	Turkey	2019	(116)
	Investigation of the epidemiological situation of BVD virus using serological and molecular biology tests.	Camel	BVD virus was found in camels transported from Sudan to Egypt.	Egypt	2018	(117)
Bluetongue (BT) virus infection	Investigation of seroprevalence and associated risk factors of BTV infection	Dromedary Camel	High prevalence of BT virus in desert and savanna as compared to the arid area.	Sudan	2017	(118)
	To determine the proportion of BTV-specific IgG antibodies in camels.	Dromedary Camel	Camels in Kassala State have a high prevalence (78.6%) of IgG antibodies against BTV, according to this study.	Sudan	2021	(55)
	To determine the proportion and distribution of sera antibodies to BT virus in various farm animals in distinct Saudi Arabian locations using competitive ELISA	Sheep, Goats, Cattle and Camel	Positive animals in all of the areas studied, indicating that serological evidence of virus exposure was widespread across the country	Saudi Arabia	2012	(119)
MERS infection	Investigation of MERS Coronavirus dissemination from camel to human	Dromedary camel	Whole genomic sequences of camel and human collected isolates were identical.	Saudi Arabia	2014	(72)
	Serological survey to determine the MERS-Coronavirus antibodies	Dromedaries	The prevalence of MERS- Coronavirus was higher in female camel and aged camels	Pakistan	2018	(120)
	MERS-coronavirus antibodies were tested in Bactrian and hybrid camels in Dubai	Bactrian and hybrid camels	Bactrian and hybrid camels are potential sources of MERS-CoV infection	United Arab Emirates	2020	(121)

**Table 2:** Vaccine trials conducted on camels for different viral diseases.

**Viral infection**	**Vaccine type**	**Targeted animal**	**Study finding**	**Region**	**Year**	**References**
MERS	DNA based vaccine	Dromedaries	Induce potent cellular immunity and antigen-specific neutralizing antibodies in camels	United States	2015	(122)
	A poxviral vectored vaccines	Dromedary camels	Confers mucosal immunity and reduction if the viral load in vaccinated camels	Netherlands	2015	(123)
Rift Valley fever virus	ChAdOx1	Dromedary camels	RVFV envelope glycoprotein was able to produce a successful immune response in camels	Saudi Arabia	2017	(124)
	CL13T	Camels	The vaccine induces a strong neutralizing antibody response and it was safe for use	Morocco	2016	(125)
	live attenuated RVF vaccine	Alpaca	Subcutaneous inoculation of vaccine caused meningoencephalitis In treated animals	South Africa	2018	(126)
Camelpox virus	Live Attenuated Egg-Based Camelpox Vaccine	Camelus bactrianus+ Camelus dromedaries	Initially, Virus neutralizing titer was high 1 month of post-vaccination but it significantly decreased after 12 months of vaccination. The vaccine was safe for use.	Republic of Kazakhstan	2021	(15)
	CMLV/115	Camels	No adverse reactions post-vaccination, vaccine have the potential to control viral load.	Sudan	2014	(17)
Peste Des Petits Ruminants Virus	Capri poxvirus recombinant vaccine	Goats	This vaccine recombinant Capri poxvirus expresses the PPR F protein that can protect goats against PPR and capripox infection	UK	2003	(127)
BVDV	Inactivated vaccine	Mice and camels	The vaccine was safe and effective as a one-shot vaccine to reduce enterotoxaemia and BVD infections in camel calves	Egypt	2021	(128)

## Conclusion

Camels are the known reservoirs and carriers of multiple viruses. Trading camels and their products, such as milk and meat, has enhanced the risk of virus transmission to other animals and humans. Several research studies have identified deadly viruses in camels through serological testing. Diagnostic testing depends on the clinical symptoms and signs of the viral infection. There is a need for effective vaccines to minimize the spread of viral infection. Although researchers have investigated using viral vaccines in many animal species, less information is available for camels. We recommend that future studies focus on designing effective vaccines for camels. Moreover, as camels are also carriers of coronavirus strains, they can produce a strong immune response against recurring coronavirus infections. Camels can act as hosts for vaccine trials against viral infections because the vaccine will likely be easy to design and can stop the transmission of viruses from animals to humans.

## Author contributions

All authors have shared in all aspects and read and agreed to the published version of the manuscript.

## Funding

This work was supported by the Deanship of Scientific Research, Vice Presidency for Graduate Studies and Scientific Research, King Faisal University, Saudi Arabia (Project No. GRANT222).

## Conflict of interest

The authors declare that the research was conducted in the absence of any commercial or financial relationships that could be construed as a potential conflict of interest.

## Publisher's note

All claims expressed in this article are solely those of the authors and do not necessarily represent those of their affiliated organizations, or those of the publisher, the editors and the reviewers. Any product that may be evaluated in this article, or claim that may be made by its manufacturer, is not guaranteed or endorsed by the publisher.
